# Resilience and emotional intelligence as mediators between personal values and life satisfaction among Chinese young adults

**DOI:** 10.3389/fpsyg.2024.1491566

**Published:** 2024-12-09

**Authors:** Fangyan Lv, Jingbin Tan, Dongzhe Shi, Dingguo Gao

**Affiliations:** ^1^School of Marxism, Sun Yat-sen University, Guangzhou, China; ^2^Department of Psychology, and Guangdong Provincial Key Laboratory of Social Cognitive Neuroscience and Mental Health, Sun Yat-sen University, Guangzhou, China; ^3^Department of Sports Science and Physical Education, Guangzhou Xinhua University, Guangzhou, China

**Keywords:** personal values, emotional intelligence, resilience, life satisfaction, young adults

## Abstract

**Background:**

Young adults are in the period of the formation and establishment of values. Even though previous research has revealed that personal values are important factors influencing young adults’ life satisfaction, it is still unknown when and under what circumstances values affect life satisfaction among young adults. Therefore, this study explored the relationship between personal values and life satisfaction among Chinese young adults, focusing on the mediating roles of resilience and emotional intelligence.

**Methods:**

A sample of *N* = 635 (271 male) young adults from four universities located in Guangzhou were recruited using a cross-sectional stratified sampling method. All participants completed the Revised Portrait Value Questionnaire, Emotional Intelligence Scale, Satisfaction with Life Scale, and Brief Resilience Scale.

**Results:**

Results revealed that: (1) self-transcendence (*r* = 0.29, *p* < 0.001), self-enhancement (*r* = 0.27, *p* < 0.001), openness to change (*r* = 0.22, *p* < 0.001), and conservation (*r* = 0.26, *p* < 0.001) were differentially positively associated with life satisfaction. Chain mediation analysis found that: there was a significant indirect effect for self-transcendence, via EI (ind = 0.070, *p* = 0.004, 95% CI = [0.027, 0.124]), and the sequential influence via EI and resilience suggested a moderate effect (ind = 0.024, *p* = 0.008, 95% CI = [0.009, 0.045]). For self-enhancement values exerted significantly negative indirect effects on LS via resilience (ind = −0.029, *p* = 0.034, 95% CI = [−0.060, −0.005]). Openness to change had a significant indirect impact on LS via EI (ind = 0.086, *p* < 0.001, 95% CI = [0.046, 0.133]), and the sequential influence via resilience and EI was significant (ind = 0.029, *p* = 0.001, 95% CI = [0.015, 0.050]). For conservation, the chain mediation model did not reveal any significant indirect effects via resilience or EI.

**Conclusion:**

Our findings extend the current literature on personal values and life satisfaction, highlighting the significant effects of resilience and emotional intelligence on the link between personal values and life satisfaction. Overall, this research helps young adults strengthen their resilience and emotional intelligence to increase the impact of values on life satisfaction.

## 1 Introduction

Personal values (PVs), as a core aspect of identity (Hitlin, 2003), are viewed as one of the important determining factors of life satisfaction (LS, Georgellis et al., 2009; Zalewska and Zwierzchowska, 2022). An individual’s values show relations to the culture, norms, beliefs, and values prevalent within their society. Individuals are steered by their values, which serve as the guiding doctrines shaping their lives (Sayer, 2011). Young adulthood, critical as it is to how people form and develop their values, profoundly impacts an individual’s long-term growth and social behavior. Many higher education systems worldwide place strong emphasis on the values of education held by young people, as reflected in the systems of higher education in countries including the Netherlands and Finland (Kuusisto et al., 2023). Higher education throughout China, especially ideological-political education (known as “Sizheng Education”), places great emphasis on the values of education held by young people. It is widely recognized that understanding the intrinsic PVs of youth is a critical issue. This is attributable to the integral influence of these values on shaping and predicting diverse psychological constructs among youth (Xie et al., 2022, 2023). Personal values affect cognition, emotion, motivation, and behavior, and they are vital to our emotions as well as behavioral decisions (Ahn and Reeve, 2021; Sagiv and Schwartz, 2022). Personal values are subjective reflections of individuals’ thoughts and feelings about themselves (Sagiv et al., 2017), and they remain a prevalent research topic across multiple disciplines, such as psychology and sociology (Bardi and Schwartz, 2003).

Schwartz’s value theory posits that values serve as benchmarks for individual behavior and judgment, and the influence of this theory has been profound. The theory has been refined and developed to encompass 19 PVs, categorized into 4 higher-order values (Schwartz et al., 2012). Self-transcendence emphasizes the individual’s concern for goals and interests that go beyond self-centeredness, such as unity, equality, social justice, and charity. It reflects the individual’s care for the well-being of others, as well as a sense of responsibility towards society and the natural environment. Conservation emphasizes the maintenance of social order and security, such as respect for tradition, conformity, and safety. This value orientation tends to preserve existing social structures and norms to ensure stability and order. Openness to change, this value dimension encourages innovation, freedom, independence, and acceptance of change. It reflects the individual’s openness to new ideas and different ways of life, as well as an emphasis on personal growth and self-expression. The value of self-enhancement emphasizes the pursuit of personal achievement, success, and social status. The relationships among these values are delineated by two dimensions: social focus (Self-Transcendence and Conservation) and personal focus (Openness to Change and Self-Enhancement) (Schwartz, 2017). Values with a social focus are more concerned with the interests of society and the collective, while those with a personal focus emphasize the needs and goals of the individual. Schwartz pointed out that PVs are cyclical, showing a continuum of motivation productive of different levels of abstraction in terms of their classification but must maintain the order of the ring structure of values according to a research problem (Schwartz et al., 2012, 2017). The classification has found support (Benish-Weisman et al., 2022; Daniel et al., 2022) focusing on a range of key topics, including predicting behaviors (Lee et al., 2022), exploring the relationship with well-being (Grosz et al., 2021; Sortheix and Schwartz, 2017), and the impact of culture and social events (Sagiv and Schwartz, 2022; Sortheix et al., 2019).

Many have studied how values relate to well-being (Arambewela and Hall, 2013; Bojanowska and Urbańska, 2021; Collins et al., 2024; Georgellis et al., 2009; Grosz et al., 2021; Hanel et al., 2020; Hanel et al., 2024; Joshanloo, 2021; Sortheix, 2018; Sortheix and Lönnqvist, 2014; Zalewska and Zwierzchowska, 2022). Life satisfaction refers to cognitive-dimension subjective well-being (SWB; Kong et al., 2019; Homocianu, 2024). It is a vital indicator of the quality as well as the overall state of life (Özer et al., 2016). Personal values refer to the cognitive expression of goal-seeking (Sagiv and Schwartz, 2022) and may affect people’s subjective evaluations concerning LS (Xie et al., 2022, 2023). Personal values are vital determinants of LS (Georgellis et al., 2009; Sortheix, 2018; Sortheix and Schwartz, 2017; Zalewska and Zwierzchowska, 2022). Moreover, people’s acts in pursuit of realizing values affect every aspect of their lives. Earlier research suggested that healthy values emphasizing autonomy, responsibility, and fairness—such as universalism, self-direction, and benevolence—are linked with needs for growth and are positively associated with LS, while unhealthy values, including conformity, security, and power, suggest transformations of deficiency needs (Bilsky and Schwartz, 1994). Georgellis et al. (2009) confirmed the significance of PVs, noting that they could also predict SWB. Furthermore, self-determination theory (Deci and Ryan, 1995) indicates certain values have particular importance and can predict changes in an individual’s well-being. Research has proposed two mechanisms to explain how pursuing values causally influences SWB (Sagiv and Schwartz, 2000). First, cultivating healthy growth values can bring about positive attitudes, behaviors, or perceptions, and increase SWB. For instance, those with values stressing universalism and benevolence may tend to perceive people as kind, be more tolerant of others, and help others more. Second, pursuing healthy values may satisfy intrinsic, self-actualizing needs, which lead directly to SWB. In contrast, pursuing unhealthy anxiety values, such as security and power, may require stressful self-protective activity. One study conducted in childhood has revealed that healthy growth values are positively associated with SWB, while Unhealthy anxiety values are negatively associated only with girls’ SWB (Collins et al., 2024).

One theoretical model suggests that values’ relation to SWB depends on an interplay of growth versus self-protection orientation and a focus on personal versus social values (Sortheix and Schwartz, 2017). Sortheix and Schwartz posited that a growth orientation, like a personal focus, may boost SWB. However, a social focus, like a self-protective, anxiety-control orientation, undermines SWB. Consequently, valuing openness to change (combining a growth orientation with a personal focus) is positive in its correlation with SWB. In contrast, valuing conservation (combining a self-protective orientation with a social focus) has a negative relation to SWB. Combinations drawing orientation together with focus, which underlie self-transcendence as well as self-enhancement values, may import a balance of opposing influences on SWB. Sortheix and Schwartz’s theoretical model was substantiated by their analyses across the European Social Survey’s 35 nations. However, studies of college students in 14 countries (most of them Asian countries) have shown that self-transcendence as well as conservation may positively predict LS (Joshanloo et al., 2016), contrary to the predictions of the theoretical model. Moreover, to some extent, LS depends on individuals’ values (Hanel et al., 2020). We do not know of any reliable evidence concerning the values most important to LS. Studies of college students in 14 countries have shown that the interaction between hedonism, self-enhancement, and self-direction predicts LS (Joshanloo et al., 2016). Openness to change, like conservation and self-transcendence, has minor to moderate associations with LS, but self-enhancement remains unrelated to LS (Zalewska and Zwierzchowska, 2022). Although the findings of previous research have confirmed a direct link joining PVs and LS, inconsistencies in findings may be attributable to sociocultural differences and analytical differences across the studies (Xie et al., 2022, 2023).

The perspective of person-environment value congruence suggests that individuals have higher LS when their personal values and priorities align with those values and priorities that prevail throughout their milieu (Musiol and Boehnke, 2013; Sagiv and Schwartz, 2000; Sortheix, 2018; van Den Broeck et al., 2019). In other words, PVs is related to LS through moderation due to culture and social context. Research on 25,442 young people worldwide (aged 18–30 years) showed that young adults have higher LS if they have intrinsic values or reside in countries (Van Den Broeck et al., 2019). Influences due to value-environment fit affecting individual LS might have less prominence in cultures favoring individualism than in cultures favoring collectivism. Personal values are often contingent upon the specific circumstances encountered and are significantly influenced by the social roles individuals assume within collectivist societies; however, in individualistic cultures, there tends to be a greater degree of stability and consistency in values across various contexts (Oyserman and Lee, 2008). Nations with elevated human development index (HDI) scores show positive associations between SWB and the majority of social-focused values, but nations ranked lower on the HDI showed negative relations between those values and SWB (Sortheix and Lönnqvist, 2014). Additionally, for person-focused values, valuing openness to change had greater positive association toward SWB among nations ranked lower on the HDI, and valuing self-enhancement was related in a negative sense to SWB among nations ranked higher on the HDI.

In addition to the socioeconomic context, the cultural context is also a critical moderator. Sortheix and Schwartz (2017) suggested that in low cultural egalitarianism societies (characterized by instability, uncertainty, and selfishness), person-focused values have a stronger association with SWB. However, those valuing a social focus are more likely to encounter obstacles. Nonetheless, Van Den Broeck et al. (2019) found that the person-environment fit perspective was not supported by their analysis, with the effect of value congruence predicting LS only existing for extrinsic values, and with a small effect size. Value consistency theory refers to consistency between values relating to personal and social dimensions (Sortheix, 2018). Personal value consistency assumes that the psychological fit that indicates a relationship between individuals and the socio-culture emerges as a personal value and examines each value (Du et al., 2021; Hanel et al., 2020). Value system consistency assumes that the psychological fit is a function of the total values profile, considering all values (Ungvary et al., 2018). Although each argument has empirical support, and these contradictory arguments have been addressed in various studies (Van Den Broeck et al., 2019), there is still no clear answer as to whether value type or fit is most important. Researchers explored the impact of different values on adults’ life satisfaction, particularly based on the longitudinal data from the German Socio-Economic Panel (SOEP), proposed the view that “one size does not fit all,” suggesting that different individuals may need different “recipes” to achieve high life satisfaction. This study found that individuals who follow altruistic, family, or religious value-based “recipes” tend to have above-average long-term life satisfaction, while the materialistic value “recipe” is associated with below-average life satisfaction (Headey and Wagner, 2019). Contrary to previous study findings, no association was found between any personal values in adolescence and life satisfaction in adulthood among Japanese workers (Iida et al., 2022). One study found that the person-environment congruence in being open to change leads to lower well-being, while congruence of self-enhancement values leads to higher well-being (Hanel et al., 2020), indicating a more complex mechanism. A study investigated how personal values and life domain satisfaction differentially predict global life satisfaction across cultures, finding that personal values and life domain satisfaction are associated with global life satisfaction in varying ways across countries (Galinha et al., 2023). The findings support the person-environment congruency values perspective indicating that personal values’ predictive power of global life satisfaction varies with cultural contexts, including individualism–collectivism and developmental levels, as well as other cultural values like uncertainty avoidance and indulgence (Galinha et al., 2023).

A significant gap is the lack of empirical data from China, a country with a distinct sociocultural context that could offer unique insights into existing theory. A latent profile analysis study on the personal values of Chinese college students and the associations between these values and mental health disorders and life satisfaction was conducted using convenient sampling with 8,540 Chinese college students. The results revealed the heterogeneity of personal values among Chinese college students, with those holding socially oriented values reporting higher life satisfaction. In contrast, students with personally oriented values reported lower life satisfaction (Xie et al., 2022, 2023). The limitations of the study include a sample primarily consisting of college students from southwestern China, which may limit the generalizability of the findings to other populations. Another research explored the personality-value relationship among Chinese adolescents, which not only revealed significant correlations between the personality profiles and the value profiles, but also uncovered the interweaving influence of traditional Chinese culture, modern culture, and postmodern culture (Chen et al., 2024). According to the direct associations and congruence in values between person and environment on Sortheix and Schwartz’s (2017) model, we hypothesize that in the context of China, a social environment that encourages innovation and individual expression, valuing openness to change may have positive association toward LS (H1). Typically, conservation values are negative in their associations with LS (H2a), but in a stable and secure social environment like China, these values could satisfy the need for continuity and order, possibly resulting in a less negative or no correlation with LS (H2b). Given the high value placed on collectivism and harmony in Chinese culture, self-transcendence values might show positivity correlated to LS (H3). Self-enhancement might be negatively correlated with LS (H4), as it could lead to conflicts between individual aspirations and societal expectations.

Numerous studies on values as related to LS mainly use correlative regression analysis or moderation analysis, but few studies discuss the mediating mechanism of values affecting LS. Two cross-national studies suggested that PVs influence individuals’ desired emotions and that they would be happier when experiencing such emotions regardless of the type (Tamir et al., 2016, 2017). This finding prompts consideration of emotional intelligence (EI), a pivotal factor in emotional information processing and mental adjustment, for the mediation it may bring to the link between PVs and LS.

Emotional intelligence encompasses ability to understand and evaluate the emotional state of oneself and others and also the expression, regulation, and utilization of individuals’ own emotions (Mayer et al., 2008). One recent systematic review has indicated the important protective role of emotional intelligence in psychological well-being (Mancini et al., 2024). It has been recognized as an important contributor toward LS (Asif et al., 2022; Blasco-Belled et al., 2022; Kong et al., 2019; Urquijo et al., 2016), with meta-analyses indicating a robust positive association (*r* = 0.39) (Sánchez-Álvarez et al., 2016). Emotional intelligence was also found to have a moderate link to PVs (Athota and O’Connor, 2014; Higgs and Lichtenstein, 2011; Contreras and Cano, 2016; Coskun et al., 2021; Hoyos-Cifuentes et al., 2024; Jacobs and Wollny, 2022). It has been identified as a mediator in various psychological outcomes, including harm avoidance (Athota and O’Connor, 2014), mental health problems (Jacobs and Wollny, 2022), and LS (Szcześniak and Tułecka, 2020). Athota and O’Connor (2014) used data gathered from 209 students at universities. They tested the direct relationship as well as the indirect one linking personal attributes, EI, and human values, finding a mild to moderate association linking values with EI. Furthermore, studies of elementary students have revealed a moderate association joining EI with values (Coskun et al., 2021). In addition to clues concerning a direct relation of EI and LS with values, other evidence suggests EI as a mediator. Emotional intelligence mediates the link joining human values with harm avoidance (Athota and O’Connor, 2014), mental health problems (Jacobs and Wollny, 2022), and LS (Szcześniak and Tułecka, 2020). There is significance in the relations of EI with values (Athota and O’Connor, 2014; Higgs and Lichtenstein, 2011; Coskun et al., 2021; Jacobs and Wollny, 2022) and EI and LS (Asif et al., 2022; Blasco-Belled et al., 2022; Kong et al., 2019; Palmer et al., 2002; Urquijo et al., 2016; Xiang et al., 2021); one aim of this research lay in exploring linkages joining PVs with LS through mediation due to EI.

Moreover, resilience, or the capacity to recuperate after experiencing adversity and trauma (Smith et al., 2008), is another important contributor to psychological well-being (Bajaj and Pande, 2016; Cohn et al., 2009; Ramos-Díaz et al., 2019; Liu et al., 2022; Yan et al., 2022). Resilience has a positive association with LS (Liu et al., 2013; Wang and Kong, 2020). Neuroimaging revealed that resilience mediates an association between orbitofrontal cortex activity with LS (Kong et al., 2018). Cultural values may affect resilience; this helps in understanding which cultural values may be beneficial for overcoming adversity and promoting resilience (Morgan Consoli and Llamas, 2013; Morgan Consoli et al., 2015). Values promoting self and others’ interests, such as benevolence and universalism, contribute to prosocial cognition, motivation, and behavior, such as altruism, tolerance, empathy, and trust, which complement health self-interest (Cacioppo et al., 2011) and may benefit LS. One research examined the role of PVs on well-being and resilience in the software industry, finding that PVs were positively associated with well-being and resilience (Yürüm and Özcan-Top, 2024). Considering PVs, resilience, and LS, resilience might be an indirect path leading from PVs to LS. Nevertheless, few studies have been implemented to investigate the effect of resilience in this relationship. Therefore, another goal in our research was exploring resilience as it may mediate the correlation of PVs to LS.

Emotional intelligence and resilience are intricately linked, and both play a significant role in improving life satisfaction. Emotional intelligence is a crucial component of well-being, enabling individuals to regulate emotions effectively and maintain interpersonal relationships (Palmer et al., 2002). Resilience, on the other hand, refers to the ability to bounce back from adversity and stress, which is significantly facilitated by higher levels of emotional intelligence (Sarrionandia et al., 2018). Previous studies have indeed found that both emotional intelligence and resilience work in concert to enhance individuals’ life satisfaction (Kartol et al., 2024; Liu et al., 2013; Ramos-Díaz et al., 2019). Emotional intelligence is positively associated with resilience and life satisfaction (Blasco-Belled et al., 2020; Collado-Soler et al., 2023; Delhom et al., 2020; Schneider et al., 2013). Moreover, emotional intelligence-based interventions have been found to increase life satisfaction and resilience (Delhom et al., 2020). By enhancing emotional intelligence, individuals can potentially increase their resilience, leading to a more satisfying life. Therefore, another goal of this study was exploring the chain role of emotional intelligence and resilience as it may mediate the correlation of PVs to LS.

**Figure 1:**
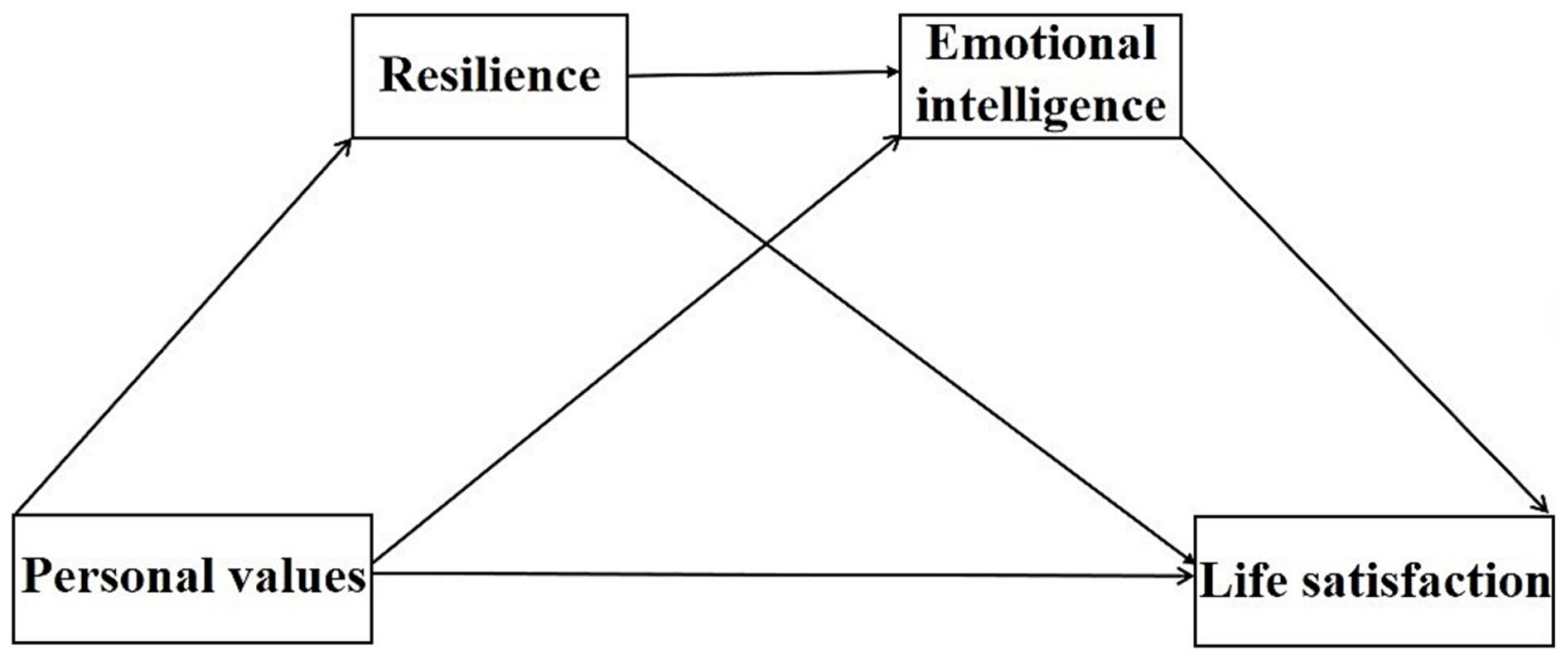
Hypothetical structure model. Personal values were accounted for on four dimensions: conservation, openness to change, self-transcendence, and self-enhancement.

In summary, we aimed to analyze what influence PVs might have when it comes to LS with resilience and EI as mediators (see Figure 1). Given the extant theory and previous research, we hypothesized that EI and resilience play mediating roles between PVs and LS, respectively (H5 and H6). Additionally, we introduced a chained mediation model positing that EI first mediates connections linking PVs with resilience, subsequently affecting LS (H7). By situating our investigation within the demographic of Chinese college students, this study seeks to offer a more profound understanding of the psychological underpinnings that connect values to well-being.

## 2 Materials and methods

### 2.1 Participants and procedure

This study employs random sampling to ensure a fair and unbiased selection of participants. In July 2022, 686 students were recruited randomly from four universities located in Guangzhou, Guangdong Province, China via the Chinese website *Wenjuanxing*^1^ within a period of one week.^2^ Invalid responses with continuous high-frequency fixed options (Curran, 2016), lie test error (DeSimone et al., 2015), and response times less than 1 min (the average time of an online answer paper) were excluded, leaving 635 remaining for analysis (effective rate of response = 92.57%). The demographic data shows that 81 (12.76%) participants were in their first year, 181 (28.50%) were sophomores, 232 (36.54%) were in their junior year, and 141 (22.21%) were in their senior year and above. Among the participants, 271 (42.68%) were males, 364 (57.32%) were females, and all were 18–29 years old (*M* = 21.23 years, SD = 1.72) (see Table 1). The first author’s Institutional Review Board granted approval for the research, ensuring strict adherence to all ethical procedures. It comprehensively addresses the ethical issues of the research, including detailed information on committee authorizations, protection declarations, and data protection regulations. All participants confirmed their informed consent, and data were collected anonymously.

**Table 1:** Demographic characteristics of the sample (*N* = 635).

Variables		*n* (%)
Gender	Male	271 (42.677%)
	Female	364 (57.323%)
Age (years)	≤20	229 (36.063%)
	21–25	394 (62.047%)
	> 25	12 (1.890%)
Years in college	≤ 1	81 (12.756%)
	1–2	181 (28.504%)
	3–4	232(36.535%)
	≥ 4	141 (22.205%)

### 2.2 Measures

#### 2.2.1 Revised portrait value questionnaire

The Revised Portrait Value Questionnaire (PVQ-RR; Schwartz and Cieciuch, 2022) comprises 57 items. Participants compared the person described in the items to themselves and evaluated, using a 6-point Likert scale (1 = not like me at all, 6 = very much like me) how similar they felt. This questionnaire was asymmetric, using two different options and four similar options, as individuals often place value on being viewed as socially acceptable. This psychological asymmetry allows for more refined discrimination (Schwartz and Cieciuch, 2016). Cronbach’s *α* for this scale was 0.95. The results of CFA in the present study demonstrated a good construct validity (χ^2^ /df = 4.086, TLI = 0.697, CFI = 0.712, RMSEA = 0.07, SRMR = 0.09).

#### 2.2.2 Satisfaction with life scale

The Satisfaction with Life Scale (SWLS; Diener et al., 1985; revised by Leung and Leung, 1992) was utilized to assess LS. There were five questions to be answered using a seven-point Likert scale. An elevated total for the items indicated more satisfaction with life. The Chinese version is reliable (Kong et al., 2015), and Cronbach’s α in this research was 0.90. The results of CFA in the present study demonstrated a good construct validity (χ^2^ /df = 8.097, TLI = 0.946, CFI = 0.968, RMSEA = 0.106, SRMR = 0.062).

#### 2.2.3 Brief resilience scale

The Brief Resilience Scale (BRS, Smith et al., 2008) includes six items evaluating the capacity for recovery after stressful events. The items were measured on a five-point Likert-type scale, and responses ranged from 1 (strongly disagree) to 5 (strongly agree). The Chinese-language BRS is reliable (Lai and Yue, 2014), and Cronbach’s α in the present study was 0.72. The results of CFA in the present study demonstrated a good construct validity (χ^2^ /df = 24.915, TLI = 0.585, CFI = 0.612, RMSEA = 0.194, SRMR = 0.137).

#### 2.2.4 Emotional intelligence scale

The Emotional Intelligence Scale (EIS; developed by Wong and Law, 2002; revised by Wong et al., 2004) was utilized. It includes four dimensions: assessing self-emotions, assessing others’ emotions, emotional use, and emotional regulation. It consists of 16 items on a seven-point Likert scale, with responses ranging from “strongly disagree” (1) to “strongly agree” (7). The Cronbach’s α in our study was 0.91, suggesting adequate reliability. Using the Mplus 7.4 to conduct confirmatory factor analysis (CFA), the results showed a good fit (χ^2^ /df = 3.409, TLI = 0.950, CFI = 0.959, RMSEA = 0.062, SRMR = 0.039).

### 2.3 Data analysis

In our research, missing data did not exceed 5% and were thus not considered in the evaluations. We computed descriptive statistics in SPSS 22.0, and mediation effects due to resilience and EI in the relationship of PVs to LS were investigated through Mplus version 7.4. To ascertain statistical significance, a bootstrapping approach with 5,000 bootstrapped resamples was adopted with a 95% confidence interval. Gender and age were found to be included in the covariate analysis controlling for any confoundment. Our primary analysis focused on factor scores of the four higher-order dimensions of PVs. For correlation coefficients relating the 10 basic values to all externals, see Appendix A.

## 3 Results

### 3.1 Descriptive statistics

Table 2 shows descriptive statistics. Results indicate that LS was positively associated with self-transcendence (*r* = 0.29, *p* < 0.001), self-enhancement (*r* = 0.27, *p* < 0.001), openness to change (*r* = 0.22, *p* < 0.001), and conservation (*r* = 0.26, *p* < 0.001). We found positive correlations between EI and self-transcendence (*r* = 0.51, *p* < 0.001), self-enhancement (*r* = 0.30, *p* < 0.001), openness to change (*r* = 0.50, *p* < 0.001), and conservation (*r* = 0.46, *p* < 0.001), and also between resilience and self-transcendence (*r* = 0.27, *p* < 0.001), self-enhancement (*r* = 0.16, *p* < 0.001), openness to change (*r* = 0.28 *p* < 0.001), and conservation (*r* = 0.20, *p* < 0.001). Finally, we found resilience (*r* = 0.48 *p* < 0.001) and EI (*r* = 0.46 *p* < 0.001) were positively related to LS. These results indicate that life satisfaction is significantly and positively correlated with individual personality traits such as self-transcendence, self-enhancement, openness to change, and conservation. Additionally, emotional intelligence and resilience are also significantly and positively correlated with life satisfaction, which may imply that these traits contribute to enhancing an individual’s life satisfaction. These findings are of great significance for understanding how individuals can achieve higher life satisfaction through various psychological resources and personality traits.

**Table 2:** Descriptive statistics and correlation.

	M	SD	1	2	3	4	5	6	7	8	9
1. Life satisfaction	22.13	6.67	–								
2. Emotional intelligence	83.79	13.60	0.46^***^	–							
3. Resilience	29.15	4.57	0.48^***^	0.52^***^	–						
4. Self-transcendence	4.61	0.68	0.29^***^	0.51^***^	0.27^***^	–					
5. Self-enhancement	4.08	0.78	0.27^***^	0.30^***^	0.16^***^	0.47^***^	–				
6. Openness to change	4.59	0.65	0.22^***^	0.50^***^	0.28^***^	0.71^***^	0.44^***^	–			
7. Conservation	4.51	0.67	0.26^***^	0.46^***^	0.20^***^	0.81^***^	0.47^***^	0.61^***^	–		
8. Age	21.23	1.72	0.05	0.04	0.10^*^	0.02	−0.02	−0.05	0.01	–	
9. Gender	—	—	−0.06	−0.03	0.01	0.02	−0.12^**^	0.07	0.07	−0.03	–

^*^p < 0.05; ^**^p < 0.01; ^***^p < 0.001.

### 3.2 Regression analyses

We carried out regression analyses to investigate relations of value orientations with LS, with a particular focus on mediation due to EI as well as resilience.

Our first regression model aimed to examine the effects of the four value orientations on LS while controlling for demographics. Demographic variables proved to be non-significant predictors of LS. Openness-to-change values had no predictive significance concerning LS (*β* = 0.004, *p* = 0.940), indicating no support for Hypothesis 1. Conservation values also had no predictive significance concerning LS (*β* = 0.035, *p* = 0.595), which is consistent with the notion that in a stable environment like China, these values may not negatively impact LS as Hypothesis 2a suggested, but rather show a neutral effect as Hypothesis 2b proposed. Self-transcendence values had significant predictive power in relation to LS (*β* = 0.187, *p* = 0.010), supporting Hypothesis 3. Self-enhancement positively predicted LS (*β* = 0.160, *p* < 0.001), contradicting Hypothesis 4.

The second regression model (Model 2) included EI and resilience as potential mediators. Adding these variables led to a significant increase in explained variance in LS, *ΔR*^2^ = 0.184. Emotional intelligence was significant in predicting LS (*β* = 0.323, *p* < 0.001), as did resilience (*β* = 0.263, *p* < 0.001). The inclusion of these mediators rendered direct effects due to self-transcendence impacting LS non-significant (*β* = 0.099, *p* = 0.130), suggesting complete mediation. Unexpectedly, openness-to-change values showed significantly negative direct effects on LS (*β* = −0.153, *p* = 0.002), which warrants further investigation. Moreover, the positive effect of self-enhancement’s impact on LS had positive significance (*β* = 0.185, *p* < 0.001).

These calculations suggest that EI and resilience may fully mediate relations of values related to self-transcendence with LS. Furthermore, the negative relationship of openness-to-change values to LS, which emerged after accounting for the mediators, indicates a potential masking effect. That is, in the absence of considering emotional intelligence and psychological resilience, the value orientation of openness-to-change may not show a negative impact on LS. These findings suggest that when assessing the impact of value orientations on LS, the mediating role of psychological resources such as emotional intelligence and psychological resilience should be taken into account (see Table 3).

**Table 3:** Life satisfaction regressions (*N* = 635).

Predictors	Model 1 Life satisfaction		Model 2 Life satisfaction	
	*β*	*p*	*β*	*p*
Age	0.050	0.182	0.029	0.396
Gender	−0.042	0.270	0.010	0.772
Self-transcendence	0.187^**^	0.010	0.099	0.130
Self-enhancement	0.160^***^	<0.001	0.185^***^	<0.001
Openness to change	0.004	0.940	−0.153^**^	0.002
Conservation	0.035	0.595	0.009	0.876
Emotion intelligence			0.323^***^	<0.001
Resilience			0.263^***^	<0.001
Adjusted R^2^	0.104		0.288	

^*^p < 0.05; ^**^p < 0.01; ^***^p < 0.001.

### 3.3 Mediation effects

**Figure 2:**
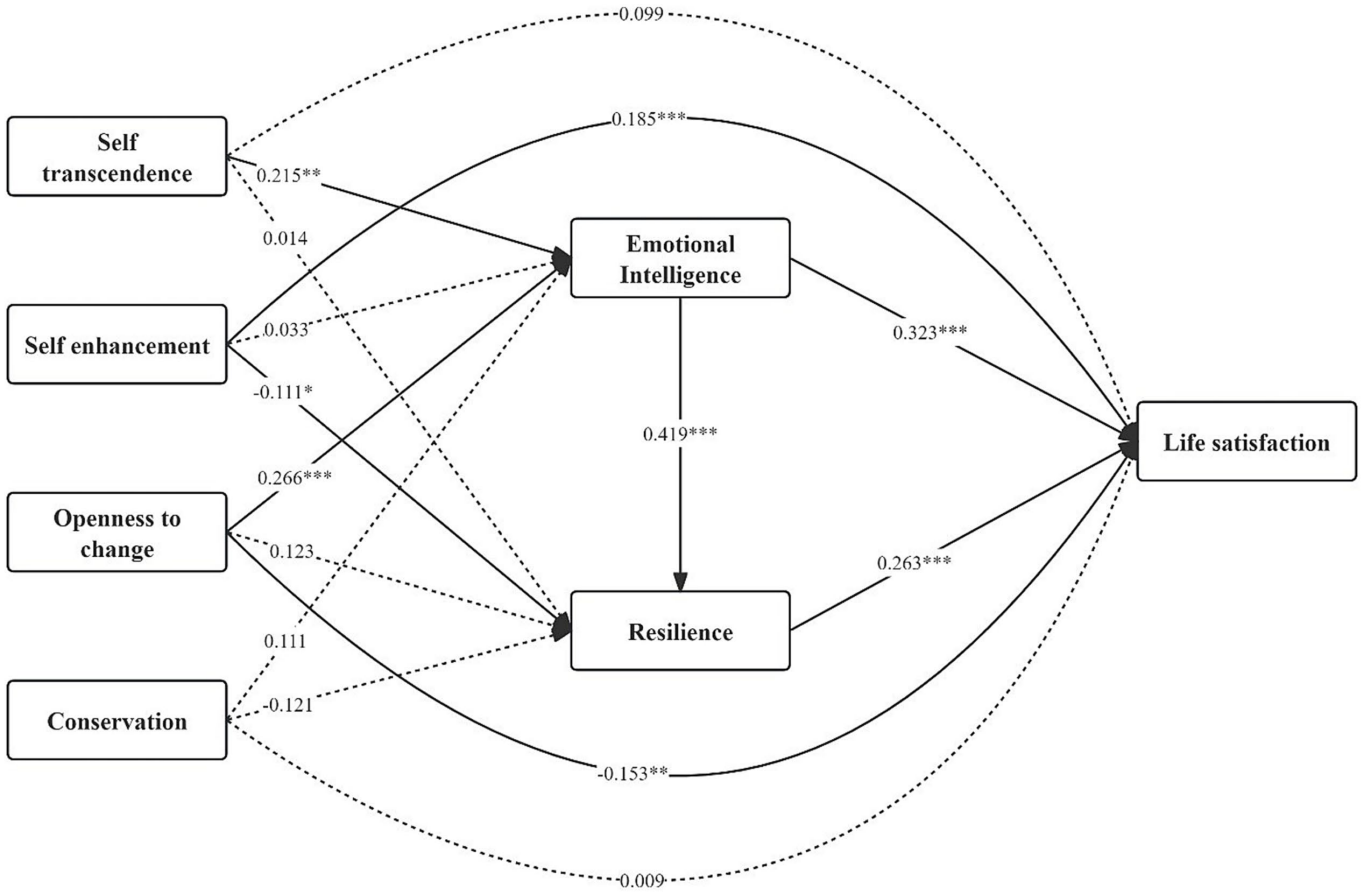
Chain-mediation model. ^*^p < 0.05, ^**^p < 0.01, ^***^p < 0.001.

A chain mediation analysis was conducted using Mplus version 7.4 to explore indirect effects associated with four higher-order dimensions of PVs concerning LS. Figure 2 depicts the mediation model’s standardized path coefficients and significance levels.

Our direct effects analysis revealed a significant positive effect of self-enhancement on LS (*β* = 0.185, *p* < 0.001). Openness to change’s effect was significantly negative (*β* = −0.153, *p* = 0.004). Self-transcendence (*β* = 0.099, *p = 0*.148), like conservation (*β* = 0.009, *p* = 0.893), did not show significant direct effects on LS. The mediators, resilience (*β* = 0.263, *p* < 0.001) and EI (*β* = 0.323, *p* < 0.001), exhibited significant direct effects on LS when we controlled for predictors and covariates.

The indirect effects analysis provided substantial evidence of mediation due to EI and resilience. For self-transcendence, there was a significant indirect effect via EI (ind = 0.070, *p* = 0.004, 95% CI = [0.027, 0.124]). This accounted for 35.7% of the overall effect due to self-transcendence concerning LS. Additionally, a significant chain mediation effect was observed, where the sequential influence of self-transcendence through EI and resilience on LS suggested a moderate effect (ind = 0.024, *p* = 0.008, 95% CI = [0.009, 0.045]), accounting for 12.2% of the overall effect. This suggests that self-transcendence values indirectly promote LS.

Conversely, self-enhancement values exerted significantly negative indirect effects on LS via resilience (ind = −0.029, *p* = 0.034, 95% CI = [−0.060, −0.005]), explaining 15.7% of the direct effects. This suggests the positive direct effect due to self-enhancement on LS was slightly diminished by its negative indirect effect.

In the case of openness to change, the sequential mediation model indicated suppression of direct effect and full mediation by resilience and EI. Despite a direct negative effect on LS, openness to change had significance in its positive indirect impact on LS via EI (ind = 0.086, *p* < 0.001, 95% CI = [0.046, 0.133]), explaining 36.6% of the total effect. Additionally, a chain mediation model revealed that the sequential influence through resilience and EI was significant (ind = 0.029, *p* = 0.001, 95% CI = [0.015, 0.050]), accounting for 19.0% of the overall effect. When all indirect effects were taken into account, the net impact of openness to change affecting LS became non-significant (*β* = −0.005, *p* = 0.925). This indicated the positive indirect pathways through resilience and EI effectively counteracted the negative direct effect, leading to a null total effect.

The sequential mediation model did not reveal any significant indirect effects due to conservation impacting LS via resilience or EI.

These results suggest that the relationship between personal value orientations and life satisfaction may be mediated by psychological resources such as emotional intelligence and psychological resilience. In particular, the value orientations of self-transcendence and openness to change indirectly affect life satisfaction through these psychological resources, while the value orientation of self-enhancement shows a complex relationship between direct and indirect effects. These findings emphasize the importance of considering the mediating role of psychological resources when assessing the impact of value orientations on an individual’s life satisfaction (see Table 4).

**Table 4:** Chained mediation model (*N* = 635).

Predictors	Mediators	Indirect effect	*p*	95%CI	P_M_
Self-transcendence	Emotional intelligence	0.07	0.004	[0.027, 0.124]	35.7%
	Resilience	0.004	0.873	[−0.044, 0.046]	–
	Emotional intelligence & resilience	0.024	0.008	[0.009, 0.045]	12.2%
Self-enhancement	Emotional intelligence	0.011	0.402	[−0.015, 0.035]	–
	Resilience	−0.029	0.034	[−0.060, −0.005]	15.7%
	Emotional intelligence & resilience	0.004	0.414	[−0.005, 0.013]	–
Openness to change	Emotional intelligence	0.086	<0.001	[0.046, 0.133]	36.6%
	Resilience	0.033	0.094	[−0.001, 0.077]	–
	Emotional intelligence & resilience	0.029	0.001	[0.015, 0.050]	19.9%
Conservation	Emotional intelligence	0.036	0.079	[−0.001, 0.081]	–
	Resilience	−0.032	0.105	[−0.076, 0.003]	–
	Emotional intelligence & resilience	0.012	0.099	[−0.001, 0.030]	–

When the main effect c’ and the indirect effect ab have the same sign, the effect ratio P_M_ is the ratio of the indirect effect to the total effect (P_M_ = ab/c); When the main effect c’ and the indirect effect ab are different signs, the effect ratio P_M_ is the absolute value of the ratio of the indirect effect to the main effect (P_M_ = |ab/c’|).

## 4 Discussion

### 4.1 Direct associations of personal values with life satisfaction

Our study investigated how four higher-order PVs relate to LS for young adults in China, emphasizing mediation roles for EI as well as resilience.

First, relation analysis indicated that each higher-order dimension of PVs positively predicted LS. The identified positive relationships of four higher-order values to LS align with theoretical frameworks positing the significance of continuity matching personal values to overall life contentment. Our four higher-order values, positive correlating EI with EI, were also positive in their correlations concerning LS, aligning with previous studies (Athota and O’Connor, 2014; Asif et al., 2022; Jacobs and Wollny, 2022; Urquijo et al., 2016). According to this study, both EI and resilience are positively related to LS. Thus, people’s raised EI and resilience yield more LS. In that respect, Kong et al. (2012, 2019) affirmed positivity in correlations linking LS with EI, and Liu et al. (2013) described positivity of correlation for resilience joined with LS. Thus, as with PVs, people with a great deal of resilience or EI have greater LS in adulthood (Temiz and Comert, 2018). This is harmonious in relation to available literature, according to which there are minuscule to moderate associations joining EI with values (Athota and O’Connor, 2014; Contreras and Cano, 2016; Coskun et al., 2021; Jacobs and Wollny, 2022). In addition, the relations of values to resilience have been explored (Morgan Consoli and Llamas, 2013; Morgan Consoli et al., 2015). These studies all explored specific value dimensions, potentially limiting their results’ interpretability and generalizability.

The regression analysis indicated that self-transcendence and -enhancement values had positive associations with LS; openness to change, like conservation values, had no significance in predicting LS. A positive effect due to self-transcendence concerning LS supports H3 and aligns with theoretical perspectives suggesting that social-focused values like self-transcendence are positive in their associations with LS among countries of high HDI featuring collectivist tendencies (Sortheix and Lönnqvist, 2014). This result shows consistency with regard to earlier studies that showed self-transcendence values contribute to lasting well-being and are negative in their associations with loneliness as well as depression (Joshanloo et al., 2016; Liu et al., 2022, 2023). A neutral effect of conservation values supports H2b, indicating that in a stable and secure social environment like China, these values may not be detrimental to LS, contrary to traditional theoretical models that associate conservation values with deficiency needs and reduced LS (Bilsky and Schwartz, 1994; Sortheix and Schwartz, 2017). This discovery contradicts traditional theories. Conventional wisdom often posits that society-focused values encourage individuals to focus more on others, which may not necessarily be beneficial to one’s sense of happiness (Sortheix and Schwartz, 2017). Yet our findings challenge this notion, demonstrating that under the umbrella of social-focused values, an individual’s happiness is enhanced. Furthermore, our results support previous studies focused on the nursing profession, which also found that society-focused values contribute to increasing the sense of well-being among nurses (Zalewska and Zwierzchowska, 2022). For nurses, their work environment can induce anxiety; hence, society-focused values have greater importance among them than personal-focused values. These values act as a safeguard with respect to stressors or dangers; moreover, they are positively correlated with LS. China, with its collectivist culture, emphasizes the importance of belonging to collective, harmonious social relations (King and Bond, 1985). Consequently, individuals are more empathetic and likely to maintain good relations in society (Xie et al., 2022, 2023).

A positive association linking LS with self-enhancement values does not support H4, challenges the theoretical model (Sortheix and Schwartz, 2017), and contradicts existing literature (Grosz et al., 2021; Liu et al., 2023), which generally proposed that pursuing power and achievement leads to stress. Previous research showed that power was significantly positively correlated with positive mental health among Russian students but negatively correlated among Chinese students (Maercker et al., 2015). These studies reported a significant, yet inconsistent, relation between self-enhancement values and LS. However, within the unique societal context of China, several factors may explain this discrepancy. First, the rapid economic development and social mobility in China may heighten the importance of self-enhancement values, aiding individuals in navigating a competitive environment and potentially enhancing LS (Sortheix and Lönnqvist, 2014). While striving for economic success can foster mental well-being, it is important to consider that it might sometimes come at a cost to one’s health (Chen et al., 2022). Second, the demographic composition of our sample—Chinese university students at a life stage that emphasizes personal effort—could amplify the positive influence of self-enhancement values on LS. For this group, self-enhancement values may be particularly influential in achieving better academic performance and enhancing future income prospects. Additionally, age appears to play a moderating role in the link joining self-enhancement values with LS, with a stronger positive association observed among young individuals (Sortheix, 2018).

The non-significant prediction of LS by openness-to-change values does not support H4 and contradicts Sortheix and Schwartz’s (2017) model, which anticipates a positive link. This discrepancy might be attributed to social collectivism in China, where individualistic aspects of openness to change may be restrained, potentially diminishing its positive impact on LS. This result is consistent with a study of Hong Kong university students (Ng and Ye, 2016) suggesting that a shared cultural context may contribute to the insignificant relationship. Furthermore, when EI and resilience were included, openness-to-change values exhibited significant, negative direct effects. Openness-to-change values might exacerbate negative hedonic experiences’ impact and affect LS (Joshanloo, 2021), while also having a positive indirect effect through EI and resilience.

### 4.2 The mediating role of emotional intelligence and resilience

Our mediation analysis aimed to uncover the mechanisms by which PVs influence LS among Chinese young adults. In this analysis, EI and resilience had significant mediation effects in relating PVs to LS.

Specifically, in relating self-transcendence values to LS, EI, and resilience played completely mediating roles through two significant paths, supporting H5 and H7. This result shows consistency with existing literature (Athota and O’Connor, 2014; Xiang et al., 2021). Individuals who endorse self-transcendence values, characterized by a focus on universal care and social harmony, often exhibit heightened empathy and emotional understanding (Ardenghi et al., 2023; Tittler et al., 2020; Xie et al., 2022, 2023). The capacity for recognizing and comprehending others’ emotions is critical when it comes to establishing or nurturing strong interpersonal relationships (Reed, 2008; Xie et al., 2022, 2023). These robust social connections have a dual benefit for LS: they directly enhance an individual’s well-being and happiness (Asif et al., 2022; Kong et al., 2012; Kong et al., 2019; Liu et al., 2022), while also providing a supportive network that can bolster resilience during experiences of stress or adversity (Jacobs and Wollny, 2022; Jibeen et al., 2018; Ramos-Díaz et al., 2019; Urquijo et al., 2016), thereby improving LS (Liu et al., 2013). Consistent with existing literature, individuals in collectivist cultural contexts tend to focus on social orientation to be more empathetic (Ardenghi et al., 2023), and greater social support (Jibeen et al., 2018) to defend against negative impacts due to stressful events (Xie et al., 2022).

Regarding conservation values, we found no significant mediation effects for EI or resilience. The non-significant impact of conservative values on LS supports an expected hypothesis. Early research posited that conservation values fall into the category of unhealthy values, representing a transformation of deficiency needs. Conservation values are generally considered to be negative factors (Bilsky and Schwartz, 1994; Sortheix and Schwartz, 2017), but in a collectivist culture like China, where public security is excellent, this societal value still has a slight positive effect, which just neutralizes the negativity. Therefore, in the mediating model, since conservative values have a low correlation with EI and resilience, to begin with, they cannot change LS on mediation paths of EI and resilience.

For self-enhancement values, only resilience showed partial mediation in relation to LS, supporting H6. The mediation effect of resilience supports available literature (Morgan Consoli and Llamas, 2013; Morgan Consoli, et al., 2015). Individuals with self-enhancement values, which stress individual success, might experience lower levels of resilience due to the stress and pressure associated with their constant pursuit of personal goals (Sortheix and Schwartz, 2017), which reduces LS (Temiz and Comert, 2018; Yan et al., 2022). Supporting previous research, people in individualistic cultural milieus tend to overly focus on personal interests and achievements, which may lead to greater psychological stress (Hanel and Wolfradt, 2016) and greater risks to mental health (Xie et al., 2022).

Interestingly, for openness-to-change values, the mediation analysis unveiled a suppression effect: a positive indirect effect due to EI and resilience concealed the negative direct effect. The positive indirect effect through EI and resilience indicates that these values could still contribute to LS by enhancing a person’s capacity for navigating social dynamics while adapting to changes. This suppression effect might be due to the individualistic aspects of openness to change, such as seeking novelty and valuing diversity, which could lead to conflict or feelings of alienation within the more collectivist and harmony-oriented Chinese culture. This may increase sensitivity to negative events and negative emotions and directly reduce LS (Joshanloo, 2021).

The culture-specific-values view holds that values converge widely in cultures, but there are essential differences between different cultures (Du et al., 2021; Oishi et al., 1998), and discoveries in one culture may not be extended to others. Evidence from previous studies using European samples supports this view (Sortheix and Lönnqvist, 2014; Van Den Broeck et al., 2019). Hence, the correlation between each higher-order value and LS may differ in different cultural environments (Xie et al., 2022). If PVs are consistent with shared values within the culture, they will likely promote LS. These findings indicate various factors may impact LS but do not work independently, rather affecting each other. This is also consistent with the notion that resilience (Morgan Consoli and Llamas, 2013), as well as EI (Jacobs and Wollny, 2022), are especially important in judging what impact PVs have on one’s LS (Georgellis et al., 2009; Sortheix, 2018; Sortheix and Schwartz, 2017; Sagiv and Schwartz, 2022).

The identified mediating roles of resilience and EI substantiate extant literature highlighting their relevance in shaping well-being outcomes. PVs, resilience, and emotional intelligence are complex but important psychological variables that affect individuals’ LS, and the relationships among them warrant further research and exploration. Several studies have examined the relationship between individual values and EI (Athota and O’Connor, 2014; Contreras and Cano, 2016; Higgs and Lichtenstein, 2011; Jacobs and Wollny, 2022; Kornilova and Chigrinova, 2014; Pérez-Fuentes et al., 2019). Our findings are consistent with previous research, indicating that values are positively correlated with EI. The inner-directed value system is positively correlated with multiple dimensions of EI. Values, as expressions of motivation, can inspire individual behavior and are linked to certain aspects of EI, especially in promoting values that enhance personal growth and social connections. Although values are relatively stable, EI is considered improvable through training and development, suggesting that enhancing EI, may indirectly influence an individual’s values and behavior. Research has revealed that PVs were positively associated with resilience (Yürüm and Özcan-Top, 2024; Zimmermann et al., 2014). Living and working in alignment with PVs can significantly enhance well-being and increase resilience (Yürüm and Özcan-Top, 2024). Research suggested that certain types of values in soldiers after military deployment were associated with resilience (Zimmermann et al., 2014). This means that individuals’ values may affect how they cope with stress and challenges, thereby affecting their level of resilience. The relationship between values and resilience may be influenced by cultural and environmental factors. Previous studies suggested that emotional intelligence, self-esteem, and resilience are significant predictors of life satisfaction among university students (Vilca-Pareja et al., 2022). Individuals with higher emotional intelligence are better equipped to deal with stress, contributing to their resilience and life satisfaction (Kartol et al., 2024). Previous research has indicated that resilience acts as a mediator in the relationship between emotional intelligence and life satisfaction (Kartol et al., 2024; Liu et al., 2013; Ramos-Díaz et al., 2019). Although no current studies have directly explored the serial mediating roles of emotional intelligence and psychological resilience between values and life satisfaction, our study’s findings confirm the mediating effects of emotional intelligence and psychological resilience between values and life satisfaction, filling a gap in the existing research.

The cultural context, deeply embedded within Chinese society, is likely to have significantly influenced the relationships observed in this study. China’s rich history, traditions, and societal norms have invariably shaped individual value systems and psychological constructs, consequently impacting the study’s outcomes. These influences can be explored through the lens of existing research on cultural psychology. Research by Hofstede (1980) and subsequent scholars has highlighted China’s strong collectivist orientation. The emphasis on familial and societal harmony, exemplified by Confucian principles, tends to nurture social-focused values such as self-transcendence or conservation. For this context, it is plausible that these values, aligned with the cultural norm of prioritizing social cohesion, manifest as significant contributors to LS. The partial mediation observed through resilience and EI can be viewed as a reflection of the collective nature of Chinese culture, where personal well-being is interwoven with harmonious interpersonal relationships.

Further, the mediation role of EI could be contextualized within the framework of guanxi, where emotional understanding and regulation enhance interpersonal relationships and, consequently, LS. The concept of “guanxi,” which pertains to personal relationships and networks, is crucial for Chinese culture. Studies (Liu et al., 2021) have established that individuals of considerable EI have greater capacities to navigate and maintain guanxi, thus contributing to their overall well-being. However, the findings regarding the mediation of self-enhancement values by EI alone might be seen as more nuanced. Chinese culture’s inherent modesty and emphasis on humility might render overt self-enhancement values less salient. As a result, EI, enabling effective self-regulation and interpersonal dynamics, could emerge as a key determinant of LS within this cultural context. Furthermore, the full mediation observed where openness-to-change values were related to LS could be attributed to China’s rapidly changing sociopolitical and economic landscape. The country’s modernization and urbanization have necessitated adaptability and openness to new experiences, and individuals who possess these values might find themselves more adept at navigating such changes, contributing to their overall LS.

In conclusion, the cultural context of Chinese society, characterized by collectivism, Confucian values, and evolving socioeconomic dynamics, is likely to shape the relationships observed in this study. Insights from existing research in cultural psychology, particularly the influence of collectivism, guanxi, and cultural norms on individual values and psychological constructs, offer a comprehensive understanding of how societal factors contribute to the mediation pathways identified in the study.

### 4.3 Limitations and implications

We have revealed a mediation effect due to resilience and EI on the link joining PVs with LS, which is a finding that could promote appreciation for values as they affect human well-being. Exploring the associations of PVs with LS, and the mediators of resilience and EI, extends and complements insights into the intrinsic mechanisms underlying relations of PVs with young adults’ LS in Chinese society and the context of the Chinese collectivist culture. Moreover, it also offers empirical support and theoretical guidance for the cultivation of college students’ values. Furthermore, it informs the implementation of psychological interventions to improve Chinese college students’ LS. Practically, there are implications suggesting interventions aimed at enhancing EI and resilience in educational and clinical settings. Culturally tailored interventions might yield the most effective outcomes, capitalizing on cultural values to bolster psychological resources.

Nevertheless, this study is not devoid of limitations. Several shortcomings should be acknowledged. Firstly, the cross-sectional nature of the data restricts causal inference. New research could utilize longitudinal studies to examine this more closely. Second, the current sample was composed of young Chinese adults. The exclusive focus on Chinese young adults limits generalizability to broader age groups or cultural contexts. Future studies need to recruit participants of different ages and cultural backgrounds. Third, by exploring the inner mechanism of the link between PVs of LS, chain mediation analyses with resilience and EI as mediators were conducted. The order of mediators between PVs and LS may lead to different results. Thus, future research should incorporate diverse samples, adopt longitudinal research designs, and consider the influence of personality traits. More research endeavors could delve deeper into the cultural dimensions influencing these relationships, potentially employing mixed-methods designs to capture qualitative insights. Exploring the interaction of value orientations with other individual differences could unveil further intricacies.

## 5 Conclusion

This study examined the influence due to PVs in LS for young adults in China, emphasizing mediation for resilience and EI. Four high-order values (self-transcendence, self-enhancement, openness to change, and conservation) were differentially positively associated with life satisfaction in China. Key findings indicate that self-transcendence and -enhancement values had positive impacts on LS; conservation and openness-to-change values did not show significant direct effects. Emotional intelligence and resilience fully mediated the relation of self-transcendence values to LS, with resilience also partially mediating self-enhancement values. A suppression effect was observed for openness-to-change values, where the positive indirect effects through EI and resilience masked the negative direct effect. Conservation did not reveal any significant indirect effects via resilience or EI. The results highlight how important the cultural milieu is for an understanding of the relations of PVs with LS. They also suggest that fostering EI and resilience could be pivotal in enhancing LS, with implications for targeted educational and psychological interventions. The findings of this study provide new insights into understanding the relationship between well-being and values across different cultural contexts, laying a foundation for future cross-cultural research. Future research should employ longitudinal designs and consider diverse cultural backgrounds to further investigate these relationships and their implications for well-being. The study’s limitations include its cross-sectional nature and the specific demographic of Chinese young adults, which may affect generalizability. Despite these limitations, we have contributed to the literature by offering insights into the mediating mechanisms linking values to LS within a collectivist culture.

## Data Availability

The original contributions presented in the study are included in the article/Supplementary material, further inquiries can be directed to the corresponding authors.

## Footnotes

^1^www.sojump.com

^2^https://www.wjx.cn/wjx/design/previewmobile.aspx?activity=162168136&s=1

## References

[ref1] Ahn J. S. Reeve J. (2021). Developmental pathways of preadolescents’ intrinsic and extrinsic values: the role of basic psychological needs satisfaction. Eur. J. Personal. 35, 151–167. doi: 10.1002/per.2274

[ref2] Arambewela R. Hall J. (2013). The interactional effects of the internal and external university environment, and the influence of personal values, on satisfaction among international postgraduate students. Stud. High. Educ. 38, 972–988. doi: 10.1080/03075079.2011.615916

[ref3] Ardenghi S. Rampoldi G. Bani M. Strepparava M. G. (2023). Personal values as early predictors of emotional and cognitive empathy among medical students. Curr. Psychol. 42, 253–261. doi: 10.1007/s12144-021-01373-8

[ref4] Asif M. Idrees M. Ghazal S. Ishaq G. (2022). Relationship of emotional intelligence and life satisfaction: mediating role of affectivity in medical students. ASEAN J. Psychiatr. 37, 135–138. doi: 10.33824/PJPR.2022.37.1.09

[ref5] Athota V. S. O’Connor P. J. (2014). How approach and avoidance constructs of personality and trait emotional intelligence predict core human values. Learn. Indi. Diff. 31, 51–58. doi: 10.1016/j.lindif.2013.12.009

[ref6] Bajaj B. Pande N. (2016). Mediating role of resilience in the impact of mindfulness on life satisfaction and affect as indices of subjective well-being. Pers. Indi. Diff. 93, 63–67. doi: 10.1016/j.paid.2015.09.005

[ref7] Bardi A. Schwartz S. H. (2003). Values and behavior: strength and structure of relations. Personal. Soc. Psychol. Bull. 29, 1207–1220. doi: 10.1177/0146167203254602 15189583

[ref9] Benish-Weisman M. Oreg S. Berson Y. (2022). The contribution of peer values to children’s values and behavior. Personal. Soc. Psychol. Bull. 48, 844–864. doi: 10.1177/01461672211020193, PMID: 34142620 PMC9121531

[ref10] Bilsky W. Schwartz S. H. (1994). Values and personality. Eur. J. Personal. 8, 163–181. doi: 10.1002/per.2410080303

[ref11] Blasco-Belled A. Rogoza R. Torrelles-Nadal C. Alsinet C. (2020). Emotional intelligence structure and its relationship with life satisfaction and happiness: new findings from the bifactor model. J. Happ. Stud. 21, 2031–2049. doi: 10.1007/s10902-019-00167-x

[ref12] Blasco-Belled A. Rogoza R. Torrelles-Nadal C. Alsinet C. (2022). Differentiating optimists from pessimists in the prediction of emotional intelligence, happiness, and life satisfaction: a latent profile analysis. J. Happ. Stud. 23, 2371–2387. doi: 10.1007/s10902-022-00507-4

[ref13] Bojanowska A. Urbańska B. (2021). Individual values and well-being: the moderating role of personality traits. Int. J. Psychol. 56, 698–709. doi: 10.1002/ijop.12751, PMID: 33751580 PMC8451848

[ref14] Cacioppo J. T. Reis H. T. Zautra A. J. (2011). Social resilience: the value of social fitness with an application to the military. Am. Psychol. 66, 43–51. doi: 10.1037/a0021419, PMID: 21219047

[ref15] Chen E. Brody G. H. Miller G. E. (2022). What are the health consequences of upward mobility? Ann. Rev. Psychol. 73, 599–628. doi: 10.1146/annurev-psych-033020-122814, PMID: 34579546 PMC10142907

[ref16] Chen Y. Fan C. Guo Y. Gao R. Yu Y. Liu Q. (2024). A person-centered analysis of the personality-value relationships among Chinese adolescents. Curr. Psychol. 43, 28275–28291. doi: 10.1007/s12144-024-06379-6

[ref17] Cohn M. A. Fredrickson B. L. Brown S. L. Mikels J. A. Conway A. M. (2009). Happiness unpacked: positive emotions increase life satisfaction by building resilience. Emotion 9, 361–368. doi: 10.1037/a0015952, PMID: 19485613 PMC3126102

[ref18] Collado-Soler R. Trigueros R. Aguilar-Parra J. M. Navarro N. (2023). Emotional intelligence and resilience outcomes in adolescent period, is knowledge really strength? Psychol. Res. Behav. Manage. 16, 1365–1378. doi: 10.2147/PRBM.S383296, PMID: 37124076 PMC10132289

[ref19] Collins P. R. Sneddon J. Lee J. A. (2024). Personal values, subjective wellbeing, and the effects of perceived social support in childhood: a pre-registered study. Eur. J. Psychol. Edu. 39, 3537–3560. doi: 10.1007/s10212-024-00800-1

[ref20] Contreras L. Cano M. C. (2016). Social competence and child-to-parent violence: analyzing the role of the emotional intelligence, social attitudes, and personal values. Deviant Behav. 37, 115–125. doi: 10.1080/01639625.2014.983024

[ref21] Coskun K. Kalin O. U. Aydemir A. (2021). Is emotional intelligence correlated with values among primary schoolers? SAGE Open 11:21582440211020747. doi: 10.1177/21582440211020747

[ref22] Curran P. G. (2016). Methods for the detection of carelessly invalid responses in survey data. J. Exp. Soc. Psychol. 66, 4–19. doi: 10.1016/j.jesp.2015.07.006

[ref23] Daniel E. Bardi A. Fischer R. Benish-Weisman M. Lee J. A. (2022). Changes in personal values in pandemic times. Soc. Psychol. Personal. Sci. 13, 572–582. doi: 10.1177/19485506211024026

[ref24] Deci E. L. Ryan R. M. (1995). Human autonomy: The basis for true self-esteem. In Efficacy, agency, and self-esteem. (ed.) Kernis M. H., (New York: Plenum Press), pp. 31–49.

[ref25] Delhom I. Satorres E. Meléndez J. C. (2020). Can we improve emotional skills in older adults? Emotional intelligence, life satisfaction, and resilience. Psychosoc. Interv. 29, 133–139. doi: 10.5093/pi2020a8

[ref26] DeSimone J. A. Harms P. D. DeSimone A. J. (2015). Best practice recommendations for data screening. J. Organ. Behav. 36, 171–181. doi: 10.1002/job.1962

[ref27] Diener E. D. Emmons R. A. Larsen R. J. Griffin S. (1985). The satisfaction with life scale. J. Pers. Assess. 49, 71–75. doi: 10.1207/s15327752jpa4901_13 16367493

[ref28] Du H. Götz F. M. Chen A. Rentfrow P. J. (2021). Revisiting values and self-esteem: a large-scale study in the United States. Eur. J. Personal. 37, 3–19. doi: 10.1177/08902070211038805

[ref29] Galinha I. C. Oishi S. Pereira C. Wirtz D. (2023). Personal values and life domain satisfaction predict global life satisfaction differently across cultures. J. Soc. Pers. Relation. 40, 3319–3343. doi: 10.1177/02654075231173157

[ref30] Georgellis Y. Tsitsianis N. Yin Y. P. (2009). Personal values as mitigating factors in the link between income and life satisfaction: evidence from the European social survey. Soc. Indic. Res. 91, 329–344. doi: 10.1007/s11205-008-9344-2

[ref31] Grosz M. P. Schwartz S. H. Lechner C. M. (2021). The longitudinal interplay between personal values and subjective well-being: a registered report. Eur. J. Personal. 35, 881–897. doi: 10.1177/08902070211012923

[ref32] Hanel P. H. P. Tunç H. Bhasin D. Litzellachner L. F. Maio G. R. (2024). Value fulfillment and well-being: clarifying directions over time. J. Pers. 92, 1037–1049. doi: 10.1111/jopy.12869, PMID: 37501351

[ref33] Hanel P. H. P. Wolfradt U. (2016). The ‘dark side’ of personal values: relations to clinical constructs and their implications. Pers. Indi. Diff. 97, 140–145. doi: 10.1016/j.paid.2016.03.045

[ref34] Hanel P. H. P. Wolfradt U. Wolf L. J. Coelho G. L. H. Maio G. R. (2020). Well-being as a function of person-country fit in human values. Nat. Commun. 11, 5150–5159. doi: 10.1038/s41467-020-18831-9, PMID: 33051452 PMC7554046

[ref35] Headey B. Wagner G. G. (2019). One size does not fit all: alternative values-based ‘recipes’ for life satisfaction. Soc. Indi. Res. 145, 581–613. doi: 10.1007/s11205-019-02108-w

[ref36] Higgs M. Lichtenstein S. (2011). Is there a relationship between emotional intelligence and individual values? An exploratory study. J. Gen. Manag. 37, 65–79. doi: 10.1177/030630701103700105

[ref37] Hitlin S. (2003). Values as the core of personal identity: drawing links between two theories of self. Soc. Psychol. Q. 66:118. doi: 10.2307/1519843

[ref38] Hofstede G. (1980). Culture’s consequences: International differences in work-related values. Beverly Hills, CA: Sage.

[ref39] Homocianu D. (2024). Life satisfaction: insights from the world values survey. Societies 14:119. doi: 10.3390/soc14070119

[ref40] Hoyos-Cifuentes J. D. Fernández-Otoya F. A. Rodríguez-Gómez W. F. Bernal-Torres C. A. (2024). Emotional intelligence, human values, creation and dissemination of content on social networks by girls in countries with emerging economies. Int. J. Adolesc. Youth 29:2306886. doi: 10.1080/02673843.2024.2306886

[ref41] Iida M. Watanabe K. Yeo S. A. Yasuma N. Nishi D. Kawakami N. (2022). Association of Personal Values in adolescence with subjective health status, meaning in life, and life satisfaction in adulthood: a cross-sectional study with retrospective recall. Japan. Psychol. Res., 1–9. doi: 10.1111/jpr.12444

[ref42] Jacobs I. Wollny A. (2022). Personal values, trait emotional intelligence, and mental health problems. Scand. J. Psychol. 63, 155–163. doi: 10.1111/sjop.12785, PMID: 34734412

[ref43] Jibeen T. Mahfooz M. Fatima S. (2018). Spiritual transcendence and psychological adjustment: the moderating role of personality in burn patients. J. Relig. Health 57, 1618–1633. doi: 10.1007/s10943-017-0484-z, PMID: 28856506

[ref44] Joshanloo M. (2021). There is no temporal relationship between hedonic values and life satisfaction: a longitudinal study spanning 13 years. J. Rese. Pers. 93:104125. doi: 10.1016/j.jrp.2021.104125

[ref45] Joshanloo M. Rizwan M. Khilji I. A. Ferreira M. C. Poon W. C. Sundaram S.. (2016). Conceptions of happiness and life satisfaction: an exploratory study in 14 national groups. Pers. Indi. Diff. 102, 145–148. doi: 10.1016/j.paid.2016.06.065

[ref46] Kartol A. Üztemur S. Griffiths M. D. Şahin D. (2024). Exploring the interplay of emotional intelligence, psychological resilience, perceived stress, and life satisfaction: a cross-sectional study in the Turkish context. BMC Psychol. 12, 362–373. doi: 10.1186/s40359-024-01860-0, PMID: 38907343 PMC11193244

[ref47] King A. Y. Bond M. H. (1985). “The Confucian paradigm of man: a sociological view” in Chinese Culture and Mental Health. eds. Tseng W.-S. Wu D. Y. H. (Orlando, Florida: Academic Press), 29–45.

[ref9002] Kong F. Ding K. Zhao J. (2015). The relationships among gratitude, self-esteem, social support and life satisfaction among undergraduate students. J. Happiness Stud. 16, 477–489.

[ref48] Kong F. Gong X. Sajjad S. Yang K. Zhao J. (2019). How is emotional intelligence linked to life satisfaction? The mediating role of social support, positive affect and negative affect. J. Happ. Stud. 20, 2733–2745. doi: 10.1007/s10902-018-00069-4

[ref49] Kong F. Ma X. You X. Xiang Y. (2018). The resilient brain: psychological resilience mediates the effect of amplitude of low-frequency fluctuations in orbitofrontal cortex on subjective well-being in young healthy adults. Soc. Cogn. Affect. Neurosci. 13, 755–763. doi: 10.1093/scan/nsy045, PMID: 29939335 PMC6121151

[ref50] Kong F. Zhao J. You X. (2012). Emotional intelligence and life satisfaction in Chinese university students: the mediating role of self-esteem and social support. Pers. Indi. Diff. 53, 1039–1043. doi: 10.1016/j.paid.2012.07.032

[ref51] Kornilova T. Chigrinova I. (2014). Personal values, moral development, and emotional intelligence in the regulation of choice in situations that involve interpersonal interactions. Psychol. J. Higher School Econ. 11, 56–74.

[ref52] Kuusisto E. de Groot I. de Ruyter D. Schutte I. Rissanen I. (2023). Values manifested in life purposes of higher education students in the Netherlands and Finland. J. Beliefs Values 1-23, 1–23. doi: 10.1080/13617672.2023.2279866

[ref53] Lai J. C. L. Yue X. (2014). Using the brief resilience scale to assess Chinese people’s ability to bounce back from stress. SAGE Open 4:215824401455438. doi: 10.1177/2158244014554386

[ref54] Lee J. A. Bardi A. Gerrans P. Sneddon J. Van Herk H. Evers U.. (2022). Are value-behavior relations stronger than previously thought? It depends on value importance. Eur. J. Personal. 36, 133–148. doi: 10.1177/08902070211002965

[ref55] Leung J. P. Leung K. (1992). Life satisfaction, self-concept, and relationship with parents in adolescence. J. Youth Adolesc. 21, 653–665. doi: 10.1007/BF01538737 24264168

[ref56] Li M. Yang D. Ding C. Kong F. (2015). Validation of the social well-being scale in a Chinese sample and invariance across gender. Soc. Indic. Res. 121, 607–618. doi: 10.1007/s11205-014-0639-1

[ref9003] Liu P. Mo B. Yang P. Li D. Liu S. Cai D. (2023). Values mediated emotional adjustment by emotion regulation: A longitudinal study among adolescents in China. Front. Psychol. 14:1093072. 37057176 10.3389/fpsyg.2023.1093072 PMC10086131

[ref57] Liu Y. Wang Z. Lü W. (2013). Resilience and affect balance as mediators between trait emotional intelligence and life satisfaction. Pers. Indi. Diff. 54, 850–855. doi: 10.1016/j.paid.2012.12.010

[ref58] Liu X. Wang Q. Zhou Z. (2022). The association between mindfulness and resilience among university students: a meta-analysis. Sustain. For. 14:10405. doi: 10.3390/su141610405

[ref59] Liu P. Zhang Y. Ji Y. Wu S. (2021). Threat upon entry: effect of coworker ostracism on newcomers’ proactive behaviors during organizational socialization. Front. Psychol. 12:545478. doi: 10.3389/fpsyg.2021.545478, PMID: 33889103 PMC8055943

[ref60] Maercker A. Zhang X. C. Gao Z. Kochetkov Y. Lu S. Sang Z.. (2015). Personal value orientations as mediated predictors of mental health: a three-culture study of Chinese, Russian, and German university students. Int. J. Clin. Health Psychol. 15, 8–17. doi: 10.1016/j.ijchp.2014.06.001, PMID: 30487817 PMC6224790

[ref61] Mancini G. Özal Z. Biolcati R. Trombini E. Petrides K. V. (2024). Trait emotional intelligence and adolescent psychological well-being: a systematic review. Int. J. Adolesc. Youth 29:2292057. doi: 10.1080/02673843.2023.2292057

[ref62] Mayer J. D. Roberts R. D. Barsade S. G. (2008). Human abilities: emotional intelligence. Ann. Rev. Psychol. 59, 507–536. doi: 10.1146/annurev.psych.59.103006.093646 17937602

[ref63] Morgan Consoli M. L. Delucio K. Noriega E. Llamas J. (2015). Predictors of resilience and thriving among Latina/o undergraduate students. Hisp. J. Behav. Sci. 37, 304–318. doi: 10.1177/0739986315589141

[ref64] Morgan Consoli M. L. Llamas J. D. (2013). The relationship between Mexican American cultural values and resilience among Mexican American college students: a mixed methods study. J. Couns. Psychol. 60, 617–624. doi: 10.1037/a0033998, PMID: 23957770

[ref65] Musiol A. Boehnke K. (2013). Person-environment value congruence and satisfaction with life. Int. J. Humanit. Soc. Sci. 3, 57–65.

[ref9004] Ng T. K. Ye S. (2016). Human values and university life satisfaction among Hong Kong Chinese university students: A cross-lagged panel analysis. Asia Pac. Educ. Rev. 25, 453–461.

[ref66] Oishi S. Schimmack U. Diener E. Suh E. M. (1998). The measurement of values and individualism-collectivism. Personal. Soc. Psychol. Bull. 24, 1177–1189. doi: 10.1177/01461672982411005

[ref67] Oyserman D. Lee S. W. S. (2008). Does culture influence what and how we think? Effects of priming individualism and collectivism. Psychol. Bull. 134, 311–342. doi: 10.1037/0033-2909.134.2.311 18298274

[ref68] Özer E. Hamarta E. Deniz M. E. (2016). Emotional intelligence, core self-evaluation, and life satisfaction. Psychology 7, 145–153. doi: 10.4236/psych.2016.72017

[ref69] Palmer B. Donaldson C. Stough C. (2002). Emotional intelligence and life satisfaction. Pers. Indiv. Diff. 33, 1091–1100. doi: 10.1016/S0191-8869(01)00215-X

[ref70] Pérez-Fuentes M. D. C. Molero Jurado M. D. M. Barragán Martín A. B. Gazquez Linares J. J. (2019). Family functioning, emotional intelligence, and values: analysis of the relationship with aggressive behavior in adolescents. Int. J. Environ. Public Health 16, 478–492. doi: 10.3390/ijerph16030478, PMID: 30736326 PMC6388189

[ref71] Ramos-Díaz E. Rodríguez-Fernández A. Axpe I. Ferrara M. (2019). Perceived emotional intelligence and life satisfaction among adolescent students: the mediating role of resilience. J. Happ. Stud. 20, 2489–2506. doi: 10.1007/s10902-018-0058-0

[ref9005] Reed P. G. (2008). Theory of self-transcendence. Middle range theory for nursing, 3, 105–129.

[ref72] Sagiv L. Roccas S. Cieciuch J. Schwartz S. H. (2017). Personal values in human life. Nat. Hum. Behav. 1, 630–639. doi: 10.1038/s41562-017-0185-3 31024134

[ref73] Sagiv L. Schwartz S. H. (2000). Value priorities and subjective well-being: direct relations and congruity effects. Eur. J. Soc. Psychol. 30, 177–198. doi: 10.1002/(SICI)1099-0992(200003/04)30:2<177::AID-EJSP982>3.0.CO;2-Z

[ref74] Sagiv L. Schwartz S. H. (2022). Personal values across cultures. Ann. Rev. Psychol. 73, 517–546. doi: 10.1146/annurev-psych-020821-125100 34665670

[ref75] Sánchez-Álvarez N. Extremera N. Fernández-Berrocal P. (2016). The relation between emotional intelligence and subjective well-being: a meta-analytic investigation. J. Posi. Psychol. 11, 276–285. doi: 10.1080/17439760.2015.1058968

[ref76] Sarrionandia A. Ramos-Díaz E. Fernández-Lasarte O. (2018). Resilience as a mediator of emotional intelligence and perceived stress: a cross-country study. Front. Psychol. 9:2653. doi: 10.3389/fpsyg.2018.02653, PMID: 30622503 PMC6308158

[ref77] Sayer A. (2011). Why things matter to people: Social science, values and ethical life. Cambridge: Cambridge University Press.

[ref78] Schneider T. R. Lyons J. B. Khazon S. (2013). Emotional intelligence and resilience. Pers. Indi. Diff. 55, 909–914. doi: 10.1016/j.paid.2013.07.460

[ref79] Schwartz S. H. (2017). “The refined theory of basic values” in Values and behavior. eds. Roccas S. Sagiv L. (Cham: Springer), 51–72.

[ref80] Schwartz S. H. Cieciuch J. (2016). Values. In: The SAGE encyclopedia of theory in psychology. ed. Miller Jr H. L. (Thousand Oaks: SAGE Publications, Inc.

[ref81] Schwartz S. H. Cieciuch J. (2022). Measuring the refined theory of individual values in 49 cultural groups: psychometrics of the revised portrait value questionnaire. Assessment 29, 1005–1019. doi: 10.1177/1073191121998760, PMID: 33682477 PMC9131418

[ref82] Schwartz S. H. Cieciuch J. Vecchione M. Davidov E. Fischer R. Beierlein C.. (2012). Refining the theory of basic individual values. J. Pers. Soc. Psychol. 103, 663–688. doi: 10.1037/a0029393, PMID: 22823292

[ref83] Schwartz S. H. Cieciuch J. Vecchione M. Torres C. Dirilen-Gumus O. Butenko T. (2017). Value tradeoffs propel and inhibit behavior: validating the 19 refined values in four countries. Eur. J. Soc. Psychol. 47, 241–258. doi: 10.1002/ejsp.2228

[ref85] Smith B. W. Dalen J. Wiggins K. Tooley E. Christopher P. Bernard J. (2008). The brief resilience scale: assessing the ability to bounce back. Inter. J. Behav. Medi. 15, 194–200. doi: 10.1080/10705500802222972, PMID: 18696313

[ref84] Sortheix F. M. (2018). “Values and subjective well-being” in Handbook of well-being. eds. Diener E. Oishi S. Tay L. (Salt Lake City: DEF Publishers), 1–25.

[ref86] Sortheix F. M. Lönnqvist J. E. (2014). Personal value priorities and life satisfaction in Europe: the moderating role of socioeconomic development. J. Cross-Cult. Psychol. 45, 282–299. doi: 10.1177/0022022113504621

[ref87] Sortheix F. M. Parker P. D. Lechner C. M. Schwartz S. H. (2019). Changes in young Europeans’ values during the global financial crisis. Soc. Psychol. Personal. Sci. 10, 15–25. doi: 10.1177/1948550617732610

[ref88] Sortheix F. M. Schwartz S. H. (2017). Values that underlie and undermine well-being: variability across countries. Euro. J. Pers. 31, 187–201. doi: 10.1002/per.2096

[ref89] Szcześniak M. Tułecka M. (2020). Family functioning and life satisfaction: the mediatory role of emotional intelligence. Psychol. Rese. Behav. Manage. 13, 223–232. doi: 10.2147/PRBM.S240898, PMID: 32184683 PMC7061410

[ref90] Tamir M. Schwartz S. H. Cieciuch J. Riediger M. Torres C. Scollon C.. (2016). Desired emotions across cultures: a value-based account. J. Pers. Soc. Psychol. 111, 67–82. doi: 10.1037/pspp0000072, PMID: 26524003

[ref91] Tamir M. Schwartz S. H. Oishi S. Kim M. Y. (2017). The secret to happiness: feeling good or feeling right? J. Exp. Psychol. Gen. 146, 1448–1459. doi: 10.1037/xge0000303 28805442

[ref92] Temiz Z. T. Comert I. T. (2018). The relationship between life satisfaction, attachment styles, and psychological resilience in university students. Dusunen Adam J. Psychiatry Neurol. Sci. 31, 274–283. doi: 10.5350/DAJPN2018310305

[ref93] Tittler M. V. Lannin D. G. Han S. Wolf L. J. (2020). Why personal values matter: values, colorblindness, and social justice action orientation. Curr. Psychol. 41, 5075–5087. doi: 10.1007/s12144-020-01006-6

[ref94] Ungvary S. McDonald K. L. Benish-Weisman M. (2018). Identifying and distinguishing value profiles in American and Israeli adolescents. J. Res. Adolesc. 28, 294–309. doi: 10.1111/jora.12330, PMID: 28653451

[ref95] Urquijo I. Extremera N. Villa A. (2016). Emotional intelligence, life satisfaction, and psychological well-being in graduates: the mediating effect of perceived stress. Applied Res. Qual. Life 11, 1241–1252. doi: 10.1007/s11482-015-9432-9

[ref96] Van Den Broeck A. Schreurs B. Proost K. Vanderstukken A. Vansteenkiste M. (2019). I want to be a billionaire: how do extrinsic and intrinsic values influence youngsters’ well-being? Ann. Am. Acad. Pol. Soc. Sci. 682, 204–219. doi: 10.1177/0002716219831658

[ref98] Vilca-Pareja V. Ruiz L. de Somocurcio A. Delgado-Morales R. Medina Zeballos L. (2022). Emotional intelligence, resilience, and self-esteem as predictors of satisfaction with life in university students. Int. J. Environ. Res. Public Health 19:16548. doi: 10.3390/ijerph192416548, PMID: 36554428 PMC9778840

[ref99] Wang K. Kong F. (2020). Linking trait mindfulness to life satisfaction in adolescents: the mediating role of resilience and self-esteem. Child Ind. Res. 13, 321–335. doi: 10.1007/s12187-019-09698-4

[ref100] Wong C. S. Law K. S. (2002). Wong and law emotional intelligence scale. Leadersh. Q. 13, 1–32. doi: 10.1037/t07398-000

[ref101] Wong C. S. Law K. S. Wong P. M. (2004). Development and validation of a forced choice emotional intelligence measure for Chinese respondents in Hong Kong. Asia Pac. J. Manag. 21, 535–559. doi: 10.1023/B:APJM.0000048717.31261.d0

[ref102] Xiang Y. Yuan R. Zhao J. (2021). Childhood maltreatment and life satisfaction in adulthood: the mediating effect of emotional intelligence, positive affect and negative affect. J. Health Psychol. 26, 2460–2469. doi: 10.1177/1359105320914381, PMID: 32338529

[ref103] Xie J. Q. Tian Y. Hu J. Yin M. Z. Sun Y. D. Shan Y. J.. (2023). The neural correlates of value hierarchies: a prospective typology based on personal value profiles of emerging adults. Front. Psychol. 14:1224911. doi: 10.3389/fpsyg.2023.1224911, PMID: 38164257 PMC10758175

[ref104] Xie J. Q. Yin X. Q. Qiu J. Yang J. Huang Y. Y. Li M.. (2022). Latent profile analysis of personal values among Chinese college students: associations with mental health disorders and life satisfaction. Curr. Psychol. 42, 27232–27244. doi: 10.1007/s12144-022-03861-x, PMID: 36277265 PMC9575634

[ref105] Yan W. Zhang L. Li W. Kong F. (2022). How is subjective family socioeconomic status related to life satisfaction in Chinese adolescents? The mediating role of resilience, self-esteem and hope. Child Indic. Res. 15, 1565–1581. doi: 10.1007/s12187-022-09936-2

[ref106] Yürüm O. R. Özcan-Top O. (2024). The crucial role of personal values on well-being and resilience in the software industry. IEEE Softw. 41, 115–123. doi: 10.1109/MS.2024.3395059

[ref107] Zalewska A. M. Zwierzchowska M. (2022). Personality traits, personal values, and life satisfaction among polish nurses. Inter. J. Environ. Rese. Pub. Health 19:13493. doi: 10.3390/ijerph192013493, PMID: 36294073 PMC9602654

[ref108] Zimmermann P. Firnkes S. Kowalski J. T. Backus J. Siegel S. Willmund G.. (2014). Personal values in soldiers after military deployment: associations with mental health and resilience. Eur. J. Psychotraumatol. 5:22939. doi: 10.3402/ejpt.v5.22939, PMID: 24808938 PMC4012073

