# Supplementary material for: Resilience and emotional intelligence as mediators between personal values and life satisfaction among Chinese young adults
Source: Front Psychol. 2024 Dec 9;15:1491566. doi: 10.3389/fpsyg.2024.1491566 (PMC11665639; doi:10.3389/fpsyg.2024.1491566)
Supplement: Supplementary file 1 [file Table_1.DOCX]

**Appendix A**

**Table A. Correlations of ten basic values (raw scores) with external variables**

| Basic values | Life satisfaction | Emotional intelligence | Resilience | Age | Gender |
| --- | --- | --- | --- | --- | --- |
| Self-direction | 0.16^***^ | 0.48^***^ | 0.20^***^ | -0.03 | 0.06 |
| Stimulation | 0.26^***^ | 0.43^***^ | 0.24^***^ | -0.03 | 0.01 |
| Hedonism | 0.14^***^ | 0.30^***^ | 0.10^*^ | -0.07 | 0.10^**^ |
| Achievement | 0.23^***^ | 0.39^***^ | 0.11^**^ | -0.02 | -0.03 |
| Power | 0.25^***^ | 0.21^***^ | -0.03 | -0.02 | -0.14^***^ |
| Security | 0.10^**^ | 0.37^***^ | 0.06 | -.02 | -0.19^***^ |
| Conformity | 0.23^***^ | .0.38^***^ | 0.08^*^ | -.02 | 0.07 |
| Tradition | 0.38^***^ | 0.43^***^ | 0.17^***^ | 0.07 | -0.13^**^ |
| Benevolence | 0.25^***^ | 0.43^***^ | 0.11^**^ | -0.01 | -0.02 |
| Universalism | 0.27^***^ | 0.48^***^ | 0.16^***^ | 0.03 | 0.05 |

*Notes:* ^*^*p* < .05; *^**^p* < .01; ^***^*p* < .001.
